# Dynamics of One-Directional Functionally Graded Plates with Different Sizes of Microstructure: Theoretical Tolerance Modelling

**DOI:** 10.3390/ma18020328

**Published:** 2025-01-13

**Authors:** Jarosław Jędrysiak, Magda Kaźmierczak-Sobińska

**Affiliations:** Department of Structural Mechanics, Łódź University of Technology, al. Politechniki 6, 90-924 Łódź, Poland

**Keywords:** functionally graded microstructured plates, tolerance-periodic microstructure, effect of microstructure, tolerance modelling, free vibrations

## Abstract

The dynamics of thin elastic one-directional non-periodic plates are considered in this paper. The structure of these plates is, at a macro level, functionally graded along the *x*_1_-axis, but at the micro level it is non-periodic (tolerance-periodic). In the plates, the effect of a microstructure size on their behaviour can play a crucial role. The tolerance modelling method allows for this effect to be taken into account. This paper mainly proposes that tolerance modelling leads to model equations of two different tolerance models for one-directional functionally graded plates with two kinds of tolerance-periodic microstructures, i.e., (a) those having a microstructure size that is an order of the plate thickness, *d~l*, and (b) those having the plate thickness that is smaller than a microstructure size, *d* << *l*. Derived model equations are characterised by slowly varying coefficients. A subset of these coefficients is contingent on the microstructure size. The models presented herein determine formulas for both fundamental lower-order vibration frequencies and higher-order vibration frequencies, which are related to the microstructure. These models of such plates are implemented in a rudimentary example of free vibrations. Using the Ritz method, formulas of frequencies are obtained.

## 1. Introduction

**Figure 1:**
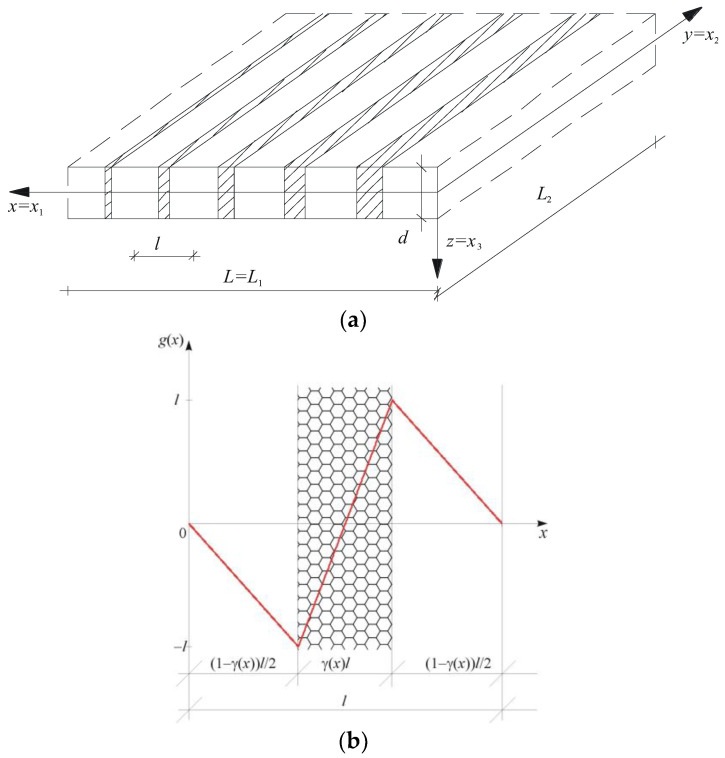
(**a**) A fragment of a thin functionally graded plate with an a-type tolerance-periodic (non-periodic) microstructure; (**b**) a cell of the plate with a saw-type fluctuation shape function.

**Figure 2:**
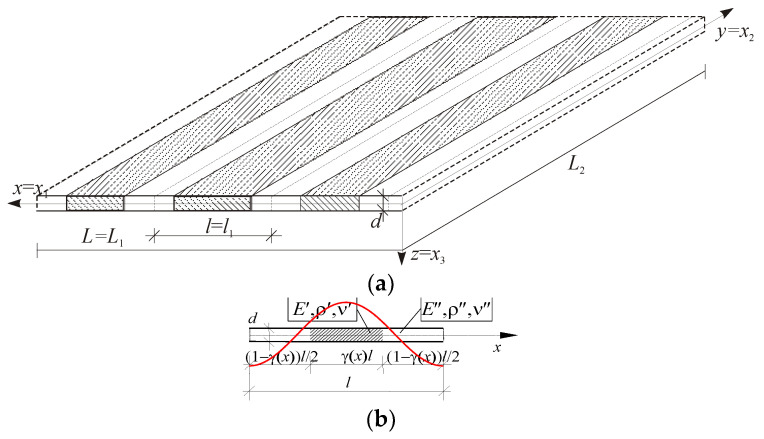
(**a**) A fragment of a thin functionally graded plate with a b-type tolerance-periodic (non-periodic) microstructure; (**b**) a cell of the plate with a fluctuation shape function.

Functionally graded (FG) plates with two kinds of tolerance-periodic (non-periodic) microstructures are considered: (a) those with an a-type tolerance-periodic microstructure, in which the size of the microstructure is of an order of the plate thickness, *d~l*, and (b) those with a b-type tolerance-periodic microstructure, having the plate thickness that is smaller than a microstructure size, *d* << *l*. These plates consist of many small elements, called cells, cf. Figure 1 and Figure 2. An example of an FG plate with an a-type microstructure is shown in Figure 1a, with a cell of the plate in Figure 1b. Conversely, Figure 2a presents a fragment of an FG plate with a b-type microstructure, and Figure 2b shows a cell of this plate.

In view of the fact that the microstructure of the plates is non-periodic along the *x*_1_ = *x* axis, a macrostructure is exhibited as functionally graded along that same axis, cf. Suresh and Mortensen [1] and Woźniak et al. [2]. These plates are frequently utilised in diverse engineering disciplines.

The dynamic problems of the thin FG plates under consideration are determined by a partial differential equation having non-continuous, highly oscillating, and tolerance-periodic coefficients. However, it is important to note that the present form of the governing equations is not an effective tool for investigating specific problems. Hence, different approximated averaging methods have been proposed.

Some averaging approaches are used for the analysis of periodic structures. Such techniques are frequently employed in the study of microstructured, functionally graded media (also of plates) [1,2]. Averaged models with the concept of effective (averaged) properties of the structure, e.g., a plate, are formulated in the framework of many of these methods. Between them, models based on asymptotic homogenisation, as in the study of Bensoussan et al. [3], can be highlighted. Effective rigidity plate theories were used to investigate periodic plates in a series of papers, e.g., in Kohn and Vogelius [4], Duvaut and Metellus [5], and Caillerie [6]. In the aforementioned models, plates are described by governing equations. These are of particular plates, with constant averaged rigidities and averaged mass densities. In order to obtain averaged properties in the context of asymptotic homogenisation, it is necessary to solve a certain boundary value problem on the periodicity cell. This problem is solved for every periodic structure. It is important to note that these procedures are usually restricted to the first approximation. However, the effect of the microstructure size usually vanishes in the model equations.

It is important to note that a range of other modelling approaches has also been proposed and developed for the purpose of describing various composite media. Several works on this type of issue can be mentioned. Some of such articles, mainly dealing with plates and shells, are indicated in this section. Homogenisation with microlocal parameters has been used to analyse periodic plates [7] and to model certain problems for a micro-periodic composite half-plane with slant lamination [8,9]. Free vibration frequencies of thick square panels made of various materials, e.g., orthotropic or hexagonal ones, were investigated in [10]. Stability problems of multi-cell thin-walled columns were analysed in [11].

Dynamic problems of three-layered, annular plates with a viscoelastic core were considered using two approximation methods—orthogonalisation and finite differences—in [12]. The buckling and post-buckling behaviours of shells of a revolution with non-classical shapes are presented in [13], using analytical–numerical models. Also, analytical–numerical methods were applied in [14,15] to analyse interesting problems of the buckling of three-layered polyethylene plates under a magnetic field. The stability of three-layered annular plates with differently damaged laminate facings under temperature fields was considered in [16], applying mainly numerical methods. In [17], computer simulations made it possible to investigate effective properties and the dynamic response of a sandwich panel made of two-faced sheets and an auxetic core. An analytical–numerical model was used to analyse dynamic problems induced by fluid flow in plates having different Poisson’s ratios in [18]. The finite element method was applied to compare the blast resistance of auxetic and non-auxetic sandwich plates in [19]. Sandwich plates with an auxetic anti-tetrachiral core have been analysed under puncture [20] and with a steady-state harmonic base motion [21] using the finite element method. The results obtained for auxetic sandwich plates were compared with the results calculated for sandwich plates having standard hexagonal honeycomb cores. Orthogonalisation and finite difference methods have been applied for annular composite plates made of layers with auxetic properties to static stability [22] and dynamic stability problems [23].

The exact strong form of governing equations for the Timoshenko–Ehrenfest beam, with geometrical nonlinearity, was formulated in [24], and then a nonlinear finite element analysis was used to obtain the weak form. Natural frequencies and mode shapes and the nonlinear free vibration were computed by applying the finite element method. In [25], the dynamic stability of a Mindlin–Reissner plate was considered, where a variational approach was used to obtain the finite element formulation of a four-node plate element. The Floquet theory and first-order approximation based on the averaging method were also applied to evaluate stability of the time-periodic system.

Many articles, which present theoretical and numerical results connected to a variety of problems concerning functionally graded media, have been published. Thermomechanical problems of functionally graded fibre-reinforced microstructure composites were considered using the higher-order theory in [26,27,28,29]. Numerous papers have successfully applied the known numerical methods for functionally graded media. For instance, the thermal analysis of composites with fibres applying the boundary element method was shown in [30]. Furthermore, the finite element method was implemented in [31] specifically for functionally graded materials. The stability analysis of functionally graded cylindrical shells demonstrated its conformity to Donnell type dynamic stability equations [32]. Meshless methods have been used in numerous papers to investigate natural frequencies of functionally graded plates [33] and sandwich beams with a functionally graded core of the structure [34]. In [35], the successful application of higher-order plate theories and a collocation method to the dynamic of functionally graded plates was presented. A GDQ solution was shown in [36] to investigate the free vibrations of shells, and higher-order deformation theories were successfully used to consider the thermomechanical problems of plates and shells with a functionally graded structure in [37,38]. In [39,40,41], the static behaviour of doubly curved functionally graded shells was analysed, while in [42] the thermal buckling of functionally graded annular plates was examined and a non-classical functionally gradient plate (FGM) model based on the modified couple stress theory was proposed. The size effect was also examined, in relation to the couple stress theory.

The free vibrations of functionally graded thick plates were investigated in [43], with consideration of the effects of normal and shear deformations. Using a higher-order normal and shear deformable plate theory, the authors of [44] analysed the vibrations of functionally graded rectangular plates. In [45], a nonlinear analysis under a shear deformation theory for functionally graded plates was presented, while in the study [46] the chaos problem pertinent to a functionally graded rectangular plate was investigated. The development of a robust formulation based on the finite element method and the GDQ technique for multilayered plates was shown in [47]. Another work on laminated composite plates can be found in [48], where the presentation of a substantial formulation of isogeometric analysis is shown. In [49], the layer-wise theory with the differential quadrature method was employed for composite plates.

In the study referenced in [50], a new low-order shell element was developed and employed for shells with functionally graded material properties. The differential quadrature method was successfully applied in the variety of issues related to functionally graded shells and plates. For instance, it was used to determine the natural frequencies of sandwich shells in [51] and to assess the dynamic stability of layered shells in [52]. In [53,54], the theory of sinusoidal shear deformation was applied to analyse the bending of piezoelectric functionally graded plates on a foundation and/or free vibrations of functionally graded composite polymer nanoplates. In addition, in [55] a semi-analytical method for investigating specific buckling, post-buckling, and dynamic problems for functionally graded thin plates was developed using the classical laminated plate theory. The same approach was also used to analyse columns made of such plates and having closed/open cross-sections (references [56,57,58]). The investigation of the free vibrations of thermally loaded functionally graded sandwich plates was conducted with the use of three-dimensional finite element modelling in [59]. Furthermore, the transient behaviour of functionally graded plates was addressed in [60] by applying a novel semi-analytical algorithm, which encompassed in-plane displacements and temperature fluctuations. The complex variable approach is used in the analysis of forces and moments acting on infinite, symmetric, functionally graded plates with a triangular hole in [61]. Another novel analytical model for sandwich structures was shown in [62], where the authors generalised the model to encompass the continuous variation in mechanical properties in the thickness direction of the structure. The individual nonlinear theory of deformation of a straight line normal to the neutral surface was applied to develop this model. In [63], an axisymmetric bending of a generalised circular sandwich plate with continuous variation in mechanical properties in the thickness direction of the core, under a concentrated force, was considered analytically with the aim of improving the shear deformation theory. Another analytical model of the elastic buckling of a sandwich plate with a functionally graded core was presented in [64] under the assumption of the nonlinear shear deformation theory of a straight normal line. In [65], the first-order shear deformation theory alongside the variational method and the Galerkin–Vlasov method were applied to analyse the dynamics of a functionally graded material porous plate resting on Winkler’s elastic foundation. Vibrations of functionally graded plates were considered in [66] using the dynamic stiffness method. In paper [67], the combination of the Jacobi–Ritz method and the multi-segment strategy, based on the higher-order shear deformation theory variational equation, were applied to propose a unified solution for the transient state vibration problem of functionally graded porous plates. Dynamical problems of nanocomposite functionally graded shells were modelled in some papers [68,69,70] using a generalised differential quadrature method. In [71], a highly accurate model was presented for the analysis of the static mechanics of pressurised annular functionally graded structures with arbitrary elastic properties along the radial direction. In the work [72], an investigation of the low-frequency vibrations of a functionally graded thin-walled cylinder in the plane state of strain was conducted. Subsequently, an asymptotic analysis of the dynamic relations in elasticity was performed across the cross-section of the cylinder, resulting in the formulation of a consistent approximate equation of motion on the mid-surface.

It is important to acknowledge that the model equations derived within the framework of the proposed modelling approaches for microstructured media tend to neglect the impact of microstructure size. On the contrary, this effect can be of significant importance in the context of vibrations in these media. As observed by Brillouin [73], a relation between macro- and microvibrations exists. To address similar issues, specific methodologies have been employed to take into account this effect. Their considerations have been extended to periodic structures in several studies, a number of which are referenced herein. In [74], the authors employed a spectral element method to analyse the characteristics of vibration band gaps in Mindlin’s periodic plates. In [75,76], the band gaps in periodic thin plates with and without damping were investigated using a centre finite difference method. Flexural wave band gaps in composite periodic plates were examined in [77] applying the differential quadrature element method.

The tolerance modelling method (or the tolerance method) is an alternative approach to the analysis of mechanical problems in microstructured media, either periodic or non-periodic. For further details, it is recommended to refer to the books by Woźniak and Wierzbicki [78], as well as Woźniak et al. (eds) [2,79]. The method has been demonstrated to be applicable in a range of problems described by differential equations with non-continuous, highly oscillating functional coefficients. The modelling procedure of the method involves substituting the exact governing equations with the averaged model equations, which are characterised by constant (or slowly varying) coefficients. It is important to note that a proportion of these coefficients are explicitly dependent on the size of the microstructure.

The tolerance modelling method enables the investigation of a range of dynamical, stability, and thermoelastic problems associated with periodic structures, as discussed in a series of articles. Some notable examples include the analysis of fluid-saturated periodic grounds, as presented in [80]. In the paper [81], the authors analysed the dynamics of periodic plane structures. An application to the vibrations of periodic medium-thickness plates was demonstrated in [82,83]. Vibrations of thin, one-directional periodic plates with a thickness smaller than the length of the periodic cell were considered in [84]. The vibrations of wavy-type periodic plates were subjected to analysis in [85]. The dynamics of thin periodic plates reinforced by stiffeners was described in [86]. The dynamics of periodic thin plates with the size of microstructure of the order of the thickness of plate was investigated in [87]. The applications of the method to various thermomechanical problems, including dynamics and stability, for thin cylindrical shells with one-directional or two-directional micro-periodic structures were presented in [88,89,90,91]. The geometrically nonlinear behaviour of thin periodically microstructured plates was investigated in [92], while the geometrically nonlinear vibrations of periodically microstructured beams were examined in [93]. A specific tolerance model for vibrations in periodic three-layered plates was proposed in [94], and the comparison of several dynamic models was discussed in [95]. The tolerance method, in conjunction with the finite difference algorithm, was employed to analyse the issue of heat transfer in biperiodic composites with third-type boundary conditions, as outlined in [96]. An investigation into the multi-scale analysis of stress distribution in thin periodic composite plates was presented in [97].

The modelling of functionally graded media with a non-periodic microstructure was also successfully achieved using the tolerance method. The thermoelasticity of transversally graded laminates, with consideration of the effect of microstructure, was analysed in [98,99]. The vibration behaviour of longitudinally graded plates was investigated in [100,101], while the stability of similar plates was examined in [102]. In [103,104], the authors analysed the dynamics of thin-walled structures having a dense system of ribs using tolerance models. The transfer of heat in composite cylindrical conductors with a non-uniform constituent distribution was described in [105,106]. Further investigations of heat conduction phenomena in laminates with functionally graded material properties and the third-type boundary conditions were conducted by [107]. The dynamics of transversally graded thin plates with a thickness smaller than the microstructure size was demonstrated in [2,108]. In the work [109], the free vibrations of thin functionally graded plates with a one-directional microstructure, having a microstructure size of the order of the plate thickness, were considered. The vibrations of functionally graded thin shells with a microstructure were presented in [110,111,112], and the dynamics and stability of them were discussed in [113]. However, it is important to note that these works do not address every issue that has been previously discussed in the literature on the tolerance modelling method for microstructured media and therefore do not represent a complete account of this field.

The principal objective of this paper is to put forth two novel tolerance models for thin, one-directional, functionally graded plates exhibiting two distinct forms of tolerance-periodic (non-periodic) microstructures: (a) an a-type tolerance-periodic microstructure, with the dimension of the microstructure of the order of the plate thickness, *d~l*; (b) a b-type tolerance-periodic microstructure, with the plate thickness smaller than the dimension of the microstructure *d* << *l*. The impact of the microstructure on the overall behaviour of the functionally graded plates with the microstructure is taken into account within the governing equations of both models. The microstructural effect of these plates can be of significant consequence in the context of dynamical issues, including free or forced vibrations. The tolerance models allow the investigation of higher-order frequencies (and higher-order vibrations, cf. Brillouin [73]) associated with the microstructure of the plate, in addition to fundamental lower frequencies (and vibrations) related to the macrostructure of the plate. Moreover, the governing equations of the asymptotic model are derived using the asymptotic homogenisation procedure, thereby eliminating the microstructural effect. This procedure is used to assess the correctness of the obtained results. The considerations presented in this paper are limited to the theoretical aspect, and as a means of illustrating the applications of the aforementioned models, formulas for the free vibration frequencies of functionally graded plate bands are derived using the Ritz method.

The main novelty of this paper is to compare and contrast two averaged tolerance models of tolerance-periodic thin plates. It is shown how the use of different formulations of one of the fundamental tolerance modelling assumptions, adapted to the nature of the microstructure of the plate under consideration, affects the obtained equations of tolerance models. All investigations are supplemented by calculation example devoted to the derivation of lower- and higher-order free vibration frequencies.

## 2. Modelling Preliminaries

Let the orthogonal Cartesian coordinate system be denoted by *Ox*_1_*x*_2_*x*_3_, and the time coordinate by *t*. Also denote *x*≡*x*_1_, *z*≡*x*_3_. Let $\Omega \equiv \{(x_{1},x_{2},z):-d/2\leq z\leq d/2,\;(x_{1},\;x_{2})\in \Pi \}$ be the region of the undeformed plate, where *d*(·) is the plate thickness; Π is the plate midplane with *L*_1_, *L*_2_ being the lengths of the plate along the *x*_1_-, *x*_2_-axis, respectively. Let subscripts *i*,*k*,*l* run over 1, 2, 3 but α,β,γ run over 1, 2. Derivatives of *x*_α_ are denoted by ∂_α_ and also ∂_α...δ_≡∂_α_...∂_δ_, and those of *t* by an overdot. Let Δ≡[−*l*/2,*l*/2]×{0} be the “basic cell” on *Ox*_1_*x*_2_, with *l* as its length dimension along the *x*_1_-axis. This length *l* is called *the microstructure parameter*.

In this paper, two types of microstructured plates are considered:

(a) Where the size of the microstructure is of an order of the plate thickness, called the *a-type microstructure*, this parameter hence satisfies the condition *d*_max_~*l* << *L*_1_;
(b) Where the plate thickness is smaller than the size of the microstructure, called the *b-type microstructure*, the condition can hence be stated as *d*_max_ << *l* << *L*_1_.

Thickness *d*(·) can be a tolerance-periodic function in *x*_1_, but elastic moduli *a_ijlm_* = *a_ijlm_*(·,*x*_2_,*z*) and a mass density ρ = ρ(·,*x*_2_,*z*) can be tolerance-periodic functions in *x*_1_ and even functions in *z*. It is assumed that the geometric and material properties of the considered plates are constant along the *x*_2_-axis direction. Using the non-zero components of the elastic moduli tensor *a*_αβγδ_, *a*_αβ33_, *a*_α3γ3_, *a*_3333_, let us denote *c*_αβγδ_≡*a*_αβγδ_ − *a*_αβ33_*a*_γδ33_(*a*_3333_)^−1^, *c*_α3γ3_≡*a*_α3γ3_ − *a*_α333_*a*_33γ3_(*a*_3333_)^−1^. Let *u_i_*(*x*_1_,*x*_2_,*x*_3_,*t*) and *i* = 1, 2, 3 be displacements of the plate along the *x_i_*-axis directions; *w*(*x*_1_,*x*_2_,*t*) = *u*(*x*_1_,*x*_2_,*t*) = *u*_3_(*x*_1_,*x*_2_,*x*_3_,*t*) be a plate deflection; and *p* be the total loadings in the *z*-axis direction.

Introducing the following denotations for the tolerance-periodic functions in *x*, the stiffnesses of the plate *b*_αβγδ_(·), the mass density μ(·), and the rotational mass inertia ϑ(·) are as follows:

(1) $$\begin{array}{l} b_{\alpha \beta \gamma \delta }(x)=\int_{-d/2}^{d/2}c_{\alpha \beta \gamma \delta }(x,y,z)z^{2}dz,\\ \mu (x)=\int_{-d/2}^{d/2}\rho (x,y,z)dz,\;\;\;\vartheta (x)=\int_{-d/2}^{d/2}\rho (x,y,z)z^{2}dz, \end{array}$$

and applying the assumptions of the Kirchhoff type plate theory to thin functionally graded plates with a microstructure, the Lagrangean Λ can be written as follows:

(2) $$\Lambda =\frac{1}{2}(\mu \dot{w}\dot{w}+\vartheta \partial_{\alpha }\dot{w}\partial_{\beta }\dot{w}\delta_{\alpha \beta })-\frac{1}{2}b_{\alpha \beta \gamma \delta }\partial_{\alpha \beta }w\partial_{\gamma \delta }w+pw$$

for which the Euler–Lagrange equation takes the following form:

(3) $$\frac{\partial }{\partial t}\frac{\partial \Lambda }{\partial \;\dot{w}}-\frac{\partial }{\partial t}\partial_{\alpha }\frac{\partial \Lambda }{\partial \;\partial_{\alpha }\dot{w}}-\partial_{\alpha \beta }\frac{\partial \Lambda }{\partial \;\partial_{\alpha \beta }w}-\frac{\partial \Lambda }{\partial \;w}=0.$$

Combining Equations (2) and (3) for deflection *w*(*x*_1_,*x*_2_), the following equation is derived:

(4) $$\partial_{\alpha \beta }(b_{\alpha \beta \gamma \delta }\partial_{\gamma \delta }w)+\mu \ddot{w}-\partial_{\alpha }(\vartheta \partial_{\beta }\ddot{w})\delta_{\alpha \beta }=p$$

which is the known fourth-order partial differential equation for thin plates and has coefficients being the non-continuous, highly oscillating, tolerance-periodic functions in *x*_1_.

## 3. Tolerance Modelling Preliminaries

### 3.1. Basic Concepts of Tolerance Method

Some basic concepts of the tolerance modelling method are introduced in the general form in the books of [2,78,79]. These concepts are shown and used in different works, e.g., in [108,109]. However, some of them are recalled here for this paper to be self-consistent.

Keeping in mind that *x* = *x*_1_, let us denote *y* = *x*_2_ and introduce an interval along the *x*-axis Γ = (0,*L*_1_).

Denoting a cell at *x*∈Γ_Δ_ by Δ(*x*)≡*x* + Δ, Γ_Δ_ = {*x*∈Γ: Δ(*x*)⊂Γ}, for an integrable function *f* the *averaging operator* is defined by the following:

(5) $$<f>(x)=l^{-1}\int_{\Delta (x)}f(\xi )d\xi ,\;x\in \Gamma ,\;\xi \in \Delta (x)$$

If a function *f* is tolerance-periodic, the averaged value obtained from (5) is a slowly varying function in *x*, but for the periodic function, this value is constant.

Let the following be denoted by $\partial^{k}f$, k=0,1,…,s, and the *k*-th gradient of function f=f(x), x∈Γ. (For problems of the considered plates, *s* = 2 or/and *s* = 1.) Let $\partial^{0}f\equiv f$ and ${\tilde{f}}^{\left(k\right)}(\cdot ,\cdot )$ be a function defined in $\bar{\Gamma }\times R^{m}$. Let us introduce the parameter δ, called *the tolerance parameter*, which is small, δ << 1. This parameter is related to and dependent on every considered problem.

*The tolerance-periodic function*, $f\in TP_{\delta }^{s}(\Gamma ,\Delta )$, is a function $f\in H^{s}(\Gamma )$, and if for k=0,1,s, the following conditions are satisfied:

$$\begin{array}{l} (i)\;(\forall x\in \Gamma )\;(\exists {\tilde{f}}^{\left(k\right)}(x,\cdot )\in H^{0}(\Gamma ))\;[||\partial^{k}f{|}_{\Gamma_{\Delta }}(\cdot )-{\tilde{f}}^{\left(k\right)}(x,\cdot )|{|}_{H^{0}(\Gamma ,\Delta )}\leq \delta ],\\ (ii)\;\int_{\Delta (\cdot )}{\tilde{f}}^{\left(k\right)}(\cdot ,\xi )d\xi \in C^{0}(\bar{\Gamma }), \end{array}$$

where function ${\tilde{f}}^{\left(k\right)}(x,\cdot )$ is *the periodic approximation of* $\partial^{k}f$ *in* Δ(x), x∈Γ, and k=0,1,s.

*The slowly varying function*, $F\in SV_{\delta }^{s}(\Gamma ,\Delta )$, is a function $F\in H^{s}(\Gamma )$ if

$$\begin{array}{l} (i)\;F\in TP_{\delta }^{s}(\Gamma ,\Delta ),\\ (ii)\;(\forall x\in \Gamma )\;[{\tilde{F}}^{\left(k\right)}(x,\cdot ){|}_{\Delta (x)}=\partial^{k}F(x),\;k=0,\ldots ,s]. \end{array}$$

*The highly oscillating function*, $\psi \in HO_{\delta }^{s}(\Gamma ,\Delta )$, is a function $\psi \in H^{s}(\Gamma )$ if

$$\begin{array}{l} (i)\;\psi \in TP_{\delta }^{s}(\Gamma ,\Delta ),\\ (ii)\;(\forall x\in \Gamma )\;[{\tilde{\psi }}^{\left(k\right)}(x,\cdot ){|}_{\Delta (x)}=\partial^{k}\tilde{\psi }(x),\;k=0,1,s],\\ (iii)\;\forall \;F\in SV_{\delta }^{s}(\Gamma ,\Delta )\;\exists f\equiv \psi F\in TP_{\delta }^{s}(\Gamma ,\Delta )\;\;{\tilde{f}}^{\left(k\right)}(x,\cdot ){|}_{\Delta (x)}=F(x)\partial^{k}\tilde{\psi }(x){|}_{\Delta (x)},\;k=1,s. \end{array}$$

Let us denote $\tilde{f}\equiv {\tilde{f}}^{\left(0\right)}$ for *k* = 0.

At this stage of investigation, two kinds of highly oscillating functions, which are *g*(·) and *h*(·), are introduced.

For FG plates with an a-type microstructure, let a highly oscillating function *g*(·) be defined on $\bar{\Gamma }$, $g\in HO_{\delta }^{1}(\Gamma ,\Delta )$, and being continuous. Gradient ∂^1^*g* is piecewise continuous and bounded. Function *g*(·) is *the fluctuation shape function* of the first kind, $FS_{\delta }^{1}(\Gamma ,\Delta )$, if it depends on *l* as a parameter and the following conditions are held:

- ∂*^k^g*∈*O*(*l^s−k^*) for *k* = 0,1, *s* = 1, ∂^0^*g*≡*g*;
- <μ*g*>(*x*)≈0 for every $x\in \Gamma_{\Delta }$.

Where *l* is the microstructure parameter, μ > 0 is a certain tolerance-periodic function.

For FG plates with a b-type microstructure let a highly oscillating function *h*(·) be defined on $\bar{\Gamma }$, $h\in HO_{\delta }^{2}(\Gamma ,\Delta )$, which is continuous together with gradient ∂^1^*h*. Gradient ∂^2^*h* is piecewise continuous and bounded. Function *h*(·) is *the fluctuation shape function* of the second kind, $FS_{\delta }^{2}(\Gamma ,\Delta )$, if it depends on *l* as a parameter and if it satisfies the conditions:

- ∂*^k^h*∈*O*(*l^s−k^*) for *k* = 0, 1, *s* = 2, ∂^0^*h*≡*h*;
- <μ*h*>(*x*)≈0 for every $x\in \Gamma_{\Delta }$.

With *l* as the microstructure parameter; μ > 0 is a certain tolerance-periodic function.

### 3.2. Tolerance Modelling Assumptions

Tolerance modelling assumptions were formulated in a general form in books [2,78,79]. In a series of papers for special problems of various structures, they were adopted applying the basic introductory concepts. Here, these assumptions are recalled for two kinds of plates under consideration.

- FG plates with an a-type microstructure (*d~l*).

The first assumption is *the micro–macro decomposition*, which is introduced as formulations of displacements of the plate:

(6) $$\begin{array}{l} u_{3}(x,y,z,t)=u(x,y,t),\\ u_{\alpha }(x,y,z,t)=-z[\partial_{\alpha }u(x,y,t)+g(x)r_{\alpha }(x,y,t)],\;\;\;x=x_{1},\;y=x_{2},\;z=x_{3},\\ u(\cdot ,y,t)\in SV_{\delta }^{2}(\Gamma ,\Delta ),r_{\alpha }(\cdot ,y,t)\in SV_{\delta }^{1}(\Gamma ,\Delta ), \end{array}$$

where functions *u*(·,*t*) and *r*_α_(·,*t*) are kinematic unknowns, named *the macrodeflection* and *the fluctuation amplitudes*, respectively. They are assumed to be slowly varying functions: the macrodeflection of the second kind, i.e., $u(\cdot ,y,t)\in SV_{\delta }^{2}(\Gamma ,\Delta )$, and the fluctuation amplitudes of the first kind, i.e., $r_{\alpha }(\cdot ,y,t)\in SV_{\delta }^{1}(\Gamma ,\Delta )$. It is assumed that function *g*(·) is the known fluctuation shape function, postulated a priori in the considered problem and describing the unknown fields (here: displacements *u*_α_, α = 1, 2) of oscillations caused by an a-type microstructure of the plate. Figure 1b shows an example of this function. This function satisfies the following restrictions:

∂*^k^g*∈*O*(*l*^1*−k*^) for *k* = 0, 1;

<μ*g*> = 0;

<μ*gg*> = 0.

The second assumption is *the tolerance averaging approximation*, in which it is assumed that terms *O*(δ) are negligibly small, e.g., for $f\in TP_{\delta }^{2}(\Gamma ,\Delta ),$ $F\in SV_{\delta }^{2}(\Gamma ,\Delta )$, $F\in SV_{\delta }^{1}(\Gamma ,\Delta )$, or $g\in FS_{\delta }^{1}(\Gamma ,\Delta ),$ in

(7) $$\begin{array}{l} <f>(x)=<\bar{f}>(x)+O(\delta ),\\ <fF>(x)=<f>(x)F(x)+O(\delta ),\\ <f\partial (gF)>(x)=<f\partial g>(x)F(x)+O(\delta ). \end{array}$$

- FG plates with b-type microstructure (*d* << *l*).

The first assumption is *the micro–macro decomposition*, which is introduced as formulations of deflection of the plate *u*:

(8) $$\begin{array}{l} u(x,y,z,t)=w(x,y,t)=W(x,y,t)+h^{A}(x)V^{A}(x,y,t),\;\;\;A=1,\ldots ,N,\\ u_{\alpha }(x,y,z,t)=-z\partial_{\alpha }W(x,y,t),\;\;\;\alpha =1,2,\\ W(\cdot ,x_{2},t),V^{A}(\cdot ,x_{2},t)\in SV_{\delta }^{2}(\Gamma ,\Delta ), \end{array}$$

where functions *W*(·,*t*) and *V^A^*(·,*t*) are new kinematic unknowns, called *the macrodeflection* and *the fluctuation amplitudes*, respectively. They are assumed to be slowly varying functions of the second kind, i.e., $W(\cdot ,y,t),\;V^{A}(\cdot ,y,t)\in SV_{\delta }^{2}(\Gamma ,\Delta )$. Functions *h^A^*(·), *A* = 1,…,*N* are assumed to be known fluctuation shape functions, a priori postulated in the considered problem and describing the unknown fields (here, the deflection) of oscillations caused by a b-type microstructure of the plate. An example of this function is shown in Figure 2b. These functions satisfy the following restrictions:

∂*^k^h^A^*∈*O*(*l*^2−^*^k^*) for *k* = 0, 1, 2;

<μ*h^A^*> = 0;

<μ*h^A^h^B^*> = 0 for *A* ≠ *B*, *A*, *B* = 1,…,*N*.

The second assumption is *the tolerance averaging approximation*, in which it is assumed that terms *O*(δ) are negligibly small, e.g., for $f\in TP_{\delta }^{2}(\Gamma ,\Delta ),$ $F\in SV_{\delta }^{2}(\Gamma ,\Delta )$, and $h^{A}\in FS_{\delta }^{2}(\Gamma ,\Delta ),$ in

(9) $$\begin{array}{l} <f>(x)=<\bar{f}>(x)+O(\delta ),\\ <fF>(x)=<f>(x)F(x)+O(\delta ),\\ <f\partial (h^{A}F)>(x)=<f\partial h^{A}>(x)F(x)+O(\delta ). \end{array}$$

## 4. Tolerance Modelling Procedure

The tolerance modelling procedure was shown in various forms in the books of [2,78,79]. In order to make this paper self-consistent, the procedure is shortly presented below, similarly to that in [2,108]. Using the Kirchhoff type plate theory assumptions with the tolerance modelling assumptions ((6) and (7) or (8) and (9)) for FG plates with an a-type or b-type microstructure and then the averaging operator (5) instead of the Lagrangean (2), one obtains the appropriate averaged Lagrangean.

### 4.1. Tolerance Modelling for FG Pates with a-Type Microstructure (d~l)

Applying the tolerance modelling for FG plates with an a-type microstructure, the tolerance-averaged Lagrangean $<\Lambda_{g}>$ is derived in the following form:

(10) $$\begin{array}{ll} <\Lambda_{g}>= & \frac{1}{2}(<\mu >\dot{u}\dot{u}+<\vartheta >\partial_{\alpha }\dot{u}\partial_{\beta }\dot{u}\delta_{\alpha \beta }+\underline{<\vartheta gg>{\dot{r}}_{\alpha }{\dot{r}}_{\beta }\delta_{\alpha \beta }})-\\ & -\frac{1}{2}[<b_{\alpha \beta \gamma \delta }>\partial_{\alpha \beta }u\partial_{\gamma \delta }u+<b_{\alpha \beta 1\delta }\partial_{1}g>\partial_{\alpha \beta }ur_{\delta }+<b_{1\beta \gamma \delta }\partial_{1}g>r_{\beta }\partial_{\gamma \delta }u+\\ & +<b_{1\beta 1\delta }\partial_{1}g\partial_{1}g>r_{\beta }r_{\delta }+\underline{<b_{2\beta 2\delta }gg>\partial_{2}r_{\beta }\partial_{2}r_{\delta }}]+<p>u. \end{array}$$

The averaged Euler–Lagrange equations for *u*(·,*x*_2_,*t*) and *r*_α_(·,*x*_2_,*t*) are obtained from the principle of stationary action applied to (10) in the following form:

(11) $$\begin{array}{l} \frac{\partial }{\partial t}\frac{\partial <\Lambda_{g}>}{\partial \;\dot{u}}-\frac{\partial }{\partial t}\partial_{\alpha }\frac{\partial <\Lambda_{g}>}{\partial \;\partial_{\alpha }\dot{u}}-\partial_{\alpha \beta }\frac{\partial <\Lambda_{g}>}{\partial \;\partial_{\alpha \beta }u}-\frac{\partial <\Lambda_{g}>}{\partial \;u}=0,\\ \frac{\partial }{\partial t}\frac{\partial <\Lambda_{g}>}{\partial \;{\dot{r}}_{\alpha }}+\partial_{2}\frac{\partial <\Lambda_{g}>}{\partial \;\partial_{2}r_{\alpha }}-\frac{\partial <\Lambda_{g}>}{\partial \;r_{\alpha }} \end{array}$$

### 4.2. Tolerance Modelling for FG Pates with b-Type Microstructure (d << l)

Similarly, by applying the tolerance modelling for FG plates with a b-type microstructure the tolerance-averaged Lagrangean $<\Lambda_{h}>$ is derived in the following form:

(12) $$\begin{array}{ll} <\Lambda_{h}>= & -\frac{1}{2}\{(<b_{\alpha \beta \gamma \delta }>\partial_{\alpha \beta }W+2<b_{\alpha \beta \gamma \delta }\partial_{\alpha \beta }h^{B}>V^{B})\partial_{\gamma \delta }W+2\underline{<b_{22\gamma \delta }h^{A}>}\partial_{22}V^{A}\partial_{\gamma \delta }W+\\ & +\underline{<b_{2222}h^{A}h^{B}>}\partial_{22}V^{A}\partial_{22}V^{B}+<b_{\alpha \beta \gamma \delta }\partial_{\alpha \beta }h^{A}\partial_{\gamma \delta }h^{B}>V^{A}V^{B}-\\ & -(\underline{<b_{1122}\partial_{11}h^{A}h^{B}>}+\underline{<b_{1122}h^{A}\partial_{11}h^{B}>}-4\underline{<b_{1122}\partial_{1}h^{A}\partial_{1}h^{B}>})\partial_{2}V^{A}\partial_{2}V^{B}-\\ & -<\mu >\dot{W}\dot{W}-\underline{<\mu h^{A}h^{B}>}{\dot{V}}^{A}{\dot{V}}^{B}-\underline{<\vartheta \partial_{\alpha }h^{A}\partial_{\beta }h^{B}>}{\dot{V}}^{A}{\dot{V}}^{B}-<\vartheta >\partial_{\alpha }\dot{W}\partial_{\beta }\dot{W}\delta_{\alpha \beta }-\\ & -\underline{<\vartheta h^{A}>}\partial_{\alpha }\dot{W}\partial_{2}{\dot{V}}^{A}-\underline{<\vartheta h^{A}h^{B}>}\partial_{2}{\dot{V}}^{A}\partial_{2}{\dot{V}}^{B}\}+<p>W+\underline{<ph^{A}>}V^{A}. \end{array}$$

The averaged Euler–Lagrange equations for *W*(·,*x*_2_,*t*) and *V^A^*(·,*x*_2_,*t*) are obtained from the principle of stationary action applied to (12) in the following form:

(13) $$\begin{array}{l} \frac{\partial }{\partial t}\frac{\partial <\Lambda_{h}>}{\partial \;\dot{W}}+\frac{\partial }{\partial t}\partial_{\alpha }\frac{\partial <\Lambda_{h}>}{\partial \;\partial_{\alpha }\dot{W}}-\partial_{\alpha \beta }\frac{\partial <\Lambda_{h}>}{\partial \;\partial_{\alpha \beta }W}-\frac{\partial <\Lambda_{h}>}{\partial \;W}=0,\\ \frac{\partial }{\partial t}\frac{\partial <\Lambda_{h}>}{\partial \;{\dot{V}}^{A}}-\partial_{22}\frac{\partial <\Lambda_{h}>}{\partial \;\partial_{22}V^{A}}+\partial_{2}\frac{\partial <\Lambda_{h}>}{\partial \;\partial_{2}V^{A}}+\frac{\partial }{\partial t}\partial_{2}\frac{\partial <\Lambda_{h}>}{\partial \;\partial_{2}{\dot{V}}^{A}}-\frac{\partial <\Lambda_{h}>}{\partial V^{A}}=0. \end{array}$$

In Lagrangeans (10) and (12), the underlined terms are dependent on the microstructure parameter *l*.

## 5. Asymptotic Modelling Approach

In order to assess the quality of results obtained by the tolerance model, an approximate asymptotic model can be applied. This model neglects the effect of microstructure size. Equations of the asymptotic model can be derived using the proper asymptotic modelling procedure, cf. [79,108,109], which is briefly sketched in the following subsections for both kinds of FG plates.

### 5.1. Tolerance Modelling for FG Pates with a-Type Microstructure (d~l)

The procedure starts with the introduction of the following: a small parameter ε∈(0,1]; an interval Δ_ε_≡[−ε*l*/2, ε*l*/2]; and ε-cell Δ_ε_(*x*)≡*x* + Δ_ε_, $x\in \bar{\Gamma }$. Then, instead of the tolerance decomposition (6), *the asymptotic decomposition* is formulated, cf. [109]. Moreover, the tolerance averaging approximation (7) is replaced by *the asymptotic approximation*, cf. [109]. Because the parameter ε tends to zero terms of an order ε (or higher), *O*(ε) can be neglected.

Let us introduce Lagrange’s function $\Lambda_{\varepsilon }=\Lambda (x,\bar{x}/\varepsilon ,\partial \partial u,u,r_{\alpha },\partial \dot{u},\dot{u})$, $\bar{x}\in \Delta_{\varepsilon }(x)$, $x\in \bar{\Gamma }$, *t*∈(*t*_0_,*t*_1_).

In the asymptotic modelling, for ε→0 function Λ_ε_ of variable $\bar{x}/\varepsilon$ $\bar{x}\in \Delta_{\varepsilon }(x)$ tends to an averaged Lagrangean Λ_0_*_g_* in the following form:

(14) $$\begin{array}{ll} \Lambda_{0g}=\frac{1}{2} & (<\mu >\dot{u}\dot{u}+<\vartheta >\partial_{\alpha }\dot{u}\partial_{\beta }\dot{u}\delta_{\alpha \beta })-\\ & -\frac{1}{2}(<b_{\alpha \beta \gamma \delta }>\partial_{\alpha \beta }u\partial_{\gamma \delta }u+2<b_{\alpha \beta 1\delta }\partial_{1}g>\partial_{\alpha \beta }ur_{\delta }+<b_{1\beta 1\delta }\partial_{1}g\partial_{1}g>r_{\beta }r_{\delta })+<p>u. \end{array}$$

The averaged asymptotic Euler–Lagrange equations for *u*(·,*x*_2_,*t*) and *r*_α_(·,*x*_2_,*t*) are obtained from the principle of stationary action applied to (14) in the following form:

(15) $$\frac{\partial }{\partial t}\frac{\partial \Lambda_{0g}}{\partial \;\dot{u}}-\frac{\partial }{\partial t}\partial_{\alpha }\frac{\partial \Lambda_{0g}}{\partial \;\partial_{\alpha }\dot{u}}-\partial_{\alpha \beta }\frac{\partial \Lambda_{0g}}{\partial \;\partial_{\alpha \beta }u}-\frac{\partial \Lambda_{0g}}{\partial \;u}=0,\;\;\;\frac{\partial \Lambda_{0g}}{\partial \;r_{\alpha }}=0.$$

The asymptotic model equations of the FG plates with an a-type microstructure can be derived by applying (14) to the averaged asymptotic Euler–Lagrange Equation (15).

### 5.2. Tolerance Modelling for FG Pates with b-Type Microstructure (d << l)

Similarly to Section 5.1, a small parameter ε∈(0,1]; an interval Δ_ε_≡[−ε*l*/2,ε*l*/2]; and ε-cell Δ_ε_(*x*)≡*x* + Δ_ε_, $x\in \bar{\Gamma }$ are introduced. Then, instead of the tolerance decomposition (8), *the asymptotic decomposition* is formulated, cf. [108]. Moreover, the tolerance averaging approximation (9) is replaced by *the asymptotic approximation*, cf. [108]. Because the parameter ε tends to zero terms of an order ε (or higher), *O*(ε) can be omitted.

Let us introduce Lagrange’s function $\Lambda_{\varepsilon }=\Lambda (x,\bar{x}/\varepsilon ,\partial \partial W,W,V^{A},\partial \dot{W},\dot{W})$, $\bar{x}\in \Delta_{\varepsilon }(x)$, $x\in \bar{\Gamma }$, *t*∈(*t*_0_,*t*_1_).

In the asymptotic modelling, for ε→0 function Λ_ε_ of variable $\bar{x}/\varepsilon$ $\bar{x}\in \Delta_{\varepsilon }(x)$ tends to an averaged Lagrangean Λ_0_*_h_* in the following form:

(16) $$\begin{array}{ll} \Lambda_{0h}=- & \frac{1}{2}\{(<b_{\alpha \beta \gamma \delta }>\partial_{\alpha \beta }W+2<b_{\alpha \beta \gamma \delta }\partial_{\alpha \beta }h^{B}>V^{B})\partial_{\gamma \delta }W+\\ & +<b_{\alpha \beta \gamma \delta }\partial_{\alpha \beta }h^{A}\partial_{\gamma \delta }h^{B}>V^{A}V^{B}-<\mu >\dot{W}\dot{W}-<\vartheta >\partial_{\alpha }\dot{W}\partial_{\beta }\dot{W}\delta_{\alpha \beta }\}+<p>W. \end{array}$$

The averaged asymptotic Euler–Lagrange equations for *W*(·,*x*_2_,*t*) and *V^A^*(·,*x*_2_,*t*) are obtained from the principle of stationary action applied to (16) in the following form:

(17) $$\frac{\partial }{\partial t}\frac{\partial \Lambda_{0h}}{\partial \;\dot{W}}-\partial_{\alpha \beta }\frac{\partial \Lambda_{0h}}{\partial \;\partial_{\alpha \beta }W}-\frac{\partial \Lambda_{0h}}{\partial \;W}=0,\;\;\;\frac{\partial \Lambda_{0h}}{\partial V^{A}}=0.$$

The asymptotic model equations of the FG plates with a b-type microstructure can be derived using the averaged asymptotic Euler–Lagrange Equation (17) with Lagrangean (16).

## 6. Governing Equations

### 6.1. Model Equations for FG Plates with a-Type Microstructure (d~l)

#### 6.1.1. Tolerance Model Equations

Combining Lagrangean (10) with Euler–Lagrange Equation (12), *the averaged equations of the tolerance model of the FG plates with an a-type microstructure* for *u*(·,*x*_2_,*t*) and *r*_α_(·,*x*_2_,*t*) are derived (α = 1, 2):

(18) $$\begin{array}{l} \partial_{\alpha \beta }(<b_{\alpha \beta \gamma \delta }>\partial_{\gamma \delta }u+<b_{\alpha \beta \gamma 1}\partial_{1}g>r_{\gamma })+<\mu >\ddot{u}-<\vartheta >\partial_{\alpha \beta }\ddot{u}\delta_{\alpha \beta }=<p>,\\ <b_{\alpha 1\gamma \delta }\partial_{1}g>\partial_{\gamma \delta }u+<b_{\alpha 1\gamma 1}\partial_{1}g\partial_{1}g>r_{\gamma }-\underline{\underline{<b_{\alpha 2\gamma 2}gg>}}\partial_{22}r_{\gamma }+\underline{<\vartheta gg>}{\ddot{r}}_{\alpha }=0. \end{array}$$

The coefficients of Equation (18) are slowly varying functions in *x*. These equations describe the effect of the microstructure size on the overall dynamic and static behaviour of the considered FG plates, as the underlined coefficients involve the microstructure parameter *l*. Double-underlined terms allow us to analyse this influence on some static issues. Equation (18) stands for the system of three differential equations for the unknowns: the macrodeflection *u* and the fluctuation amplitudes *r*_α_, α = 1, 2. These functions have to be *slowly varying functions* in *x*. For the macrodeflection *u*, two boundary conditions have to be formulated at all edges of the plate, but for the fluctuation amplitudes *r*_α_, α = 1, 2, two boundary conditions have to be formulated at both edges parallel to the microstructure of the plate.

#### 6.1.2. Asymptotic Model Equations

After combining Lagrangean (14) with Euler–Lagrange Equation (15), *the averaged equations of the asymptotic model of the FG plates with an a-type microstructure* for *u*(·,*x*_2_,*t*) and *r*_α_(·,*x*_2_,*t*) are derived (α = 1, 2):

(19) $$\begin{array}{l} \partial_{\alpha \beta }(<b_{\alpha \beta \gamma \delta }>\partial_{\gamma \delta }u+<b_{\alpha \beta \gamma 1}\partial_{1}g>r_{\gamma })+<\mu >\ddot{u}-<\vartheta >\partial_{\alpha \beta }\ddot{u}\delta_{\alpha \beta }=<p>,\\ <b_{\alpha 1\gamma \delta }\partial_{1}g>\partial_{\gamma \delta }u+<b_{\alpha 1\gamma 1}\partial_{1}g\partial_{1}g>r_{\gamma }=0. \end{array}$$

Similarly to (18), Equation (19) has coefficients being slowly varying functions in *x*. These equations neglect the effect of the microstructure size on the overall behaviour of the considered FG plates. Equation (19) stands for the system of one differential equation for the unknown macrodeflection *u* and two algebraic equations for the fluctuation amplitudes *r*_α_, α = 1, 2. These functions have to be *slowly varying functions* in *x*. For these plates, two boundary conditions have to be formulated at all edges of the plate only for the macrodeflection *u*. It can be observed that neglecting terms with the microstructure parameter *l* in Equation (18) leads directly to Equation (19).

### 6.2. Model Equations for FG Plates with b-Type Microstructure (d << l)

#### 6.2.1. Tolerance Model Equations

Combining Lagrangean (12) with Euler–Lagrange Equation (13), *the averaged equations of the tolerance model of the FG plates with a b-type microstructure* for *W*(·,*x*_2_,*t*) and *V^A^*(·,*x*_2_,*t*), *A* = 1,…, *N* are derived:

(20) $$\begin{array}{l} \partial_{\alpha \beta }(<b_{\alpha \beta \gamma \delta }>\partial_{\gamma \delta }W+<b_{\alpha \beta 11}\partial_{11}h^{A}>V^{A})+\\ +<\mu >\ddot{W}-\partial_{\alpha }(<\vartheta >\partial_{\beta }\ddot{W}\delta_{\alpha \beta })+\\ +\underline{\underline{\partial_{\alpha \beta }(<b_{\alpha \beta 22}h^{A}>\partial_{22}V^{A})}}-\underline{\partial_{2}(<\vartheta h^{A}>\partial_{2}{\ddot{V}}^{A})}=<p>,\\ <b_{\alpha \beta 11}\partial_{11}h^{A}>\partial_{\alpha \beta }W+\underline{\underline{\partial_{\alpha \beta }(<b_{\alpha \beta 22}h^{A}>\partial_{22}W)}}+\\ +<b_{1111}\partial_{11}h^{A}\partial_{11}h^{B}>V^{B}+\underline{<\mu h^{A}h^{B}>{\ddot{V}}^{B}}+\underline{<\vartheta \partial_{\alpha }h^{A}\partial_{\beta }h^{B}>{\ddot{V}}^{B}}+\\ +\underline{\underline{2(<b_{2211}\partial_{11}h^{A}h^{B}>-2<b_{2211}\partial_{1}h^{A}\partial_{1}h^{B}>)\partial_{22}V^{B}}}+\\ +\underline{\underline{\partial_{22}(<b_{2222}h^{A}h^{B}>\partial_{22}V^{B})}}-\underline{\partial_{2}(<\vartheta h^{A}>\partial_{2}\ddot{W}+<\vartheta h^{A}h^{B}>\partial_{2}{\ddot{V}}^{B})}=\underline{\underline{<ph^{A}>}}. \end{array}$$

Equation (20) also has slowly varying functional coefficients in *x*. These equations describe the effect of the microstructure size on the overall dynamic and static behaviour of the considered FG plates, as the underlined coefficients involve the microstructure parameter *l*. Double-underlined terms allow us to analyse this effect on some static issues. Equation (20) stands for the system of *N + 1* differential equations for the unknowns: the macrodeflection is *W* and the fluctuation amplitudes are *V^A^*(·,*x*_2_,*t*), *A* = 1,…, *N*. These functions have to be *slowly varying functions* in *x*. For the macrodeflection *W*, two boundary conditions have to be formulated at all edges of the plate, but for the fluctuation amplitudes *V^A^*(·,*x*_2_,*t*), *A* = 1,…, *N*, two boundary conditions have to be formulated at both edges parallel to the microstructure of the plate.

#### 6.2.2. Asymptotic Model Equations

After combining Lagrangean (16) with Euler–Lagrange Equation (17), *the averaged equations of the asymptotic model of the FG plates with a b-type microstructure* for *W*(·,*x*_2_,*t*) and *V^A^*(·,*x*_2_,*t*), *A* = 1,…, *N* are derived:

(21) $$\begin{array}{l} \partial_{\alpha \beta }(<b_{\alpha \beta \gamma \delta }>\partial_{\gamma \delta }W+<b_{\alpha \beta 11}\partial_{11}h^{A}>V^{A})+<\mu >\ddot{W}-\partial_{\alpha }(<\vartheta >\partial_{\beta }\ddot{W}\delta_{\alpha \beta })=<p>,\\ <b_{\alpha \beta 11}\partial_{11}h^{A}>\partial_{\alpha \beta }W+<b_{1111}\partial_{11}h^{A}\partial_{11}h^{B}>V^{B}=0. \end{array}$$

Similarly to (20), Equation (21) has coefficients being slowly varying functions in *x*. These equations neglect the effect of the microstructure size on the overall behaviour of the considered FG plates. Equation (21) stands for the system of one differential equation for the unknown macrodeflection *W* and *N* algebraic equations for the fluctuation amplitudes *V^A^*(·,*x*_2_,*t*), *A* = 1,…, *N*. These functions have to be *slowly varying functions* in *x*. For these plates, two boundary conditions have to be formulated at all edges of the plate only for the macrodeflection *W*. It can be observed that neglecting terms with the microstructure parameter *l* in Equation (20) leads directly to Equation (21).

## 7. An Example: Formulas of Free Vibration Frequencies for a Special FG Plate Band

### 7.1. Preliminaries

Let us consider two kinds of FG plate bands: case (1) with a span *L* along the *x*-axis, simply supported on the edges *x* = 0, *L*; case (2) with a span *L*_2_ along the *y*-axis, simply supported on the edges *y* = 0, *L*_2_. It is assumed that the plate bands are made of two component elastic isotropic materials, described by the following: Young’s moduli *E*′, *E*″, as well as mass densities ρ′, ρ″. Let the plate thickness *d* and Poisson’s ratio ν be constant; load *p* = 0 and $\partial \equiv \partial_{1},\;\bar{\partial }\equiv \partial_{2}$. Moreover, all geometrical and material properties are constant along the *y*-axis.

It is assumed that the tolerance-periodic distribution of Young’s modulus *E* and mass density is described as follows:

(22) $$E(\cdot ,\bar{x})=\rho (\cdot ,\bar{x})=\left\{\begin{array}{l} E^{\prime },\rho^{\prime },\;\;\;\mathrm{for}\;\;\bar{x}\in ((1-\gamma (x))l/2,(1+\gamma (x))l/2),\\ E^{\prime \prime },\rho^{\prime \prime },\;\;\;\mathrm{for}\;\;\bar{x}\in [0,(1-\gamma (x))l/2]\cup [(1+\gamma (x))l/2,l], \end{array}\right.$$

where γ(*x*), *x*∈[0,*L*] is a distribution function of material properties.

For *the FG plate bands with an a-type microstructure*, the form of the fluctuation shape function *g* is related to the structure of the “basic cell” shown in Figure 1b. Hence, the periodic approximation of this function can be assumed as follows:

(23) $$\tilde{g}(x,\bar{x})=\left\{\begin{array}{ll} -\frac{2}{1-\tilde{\gamma }(x)}\bar{x}, & \bar{x}\in [0,(1-\tilde{\gamma }(x))l/2],\\ \frac{2}{\tilde{\gamma }(x)}\bar{x}-\frac{l}{\tilde{\gamma }(x)}, & \bar{x}\in [(1-\tilde{\gamma }(x))l/2,(1+\tilde{\gamma }(x))l/2],\\ -\frac{2}{1-\tilde{\gamma }(x)}\bar{x}+\frac{2l}{1-\tilde{\gamma }(x)}, & \bar{x}\in [(1+\tilde{\gamma }(x))l/2,l], \end{array}\right.\;x\in [0,L],\bar{x}\in \Delta (x),$$

where $\tilde{\gamma }(x)$ is the periodic approximation of the distribution function of material properties γ(*x*).

For *the FG plate bands with a b-type microstructure*, only one fluctuation shape function is assumed, i.e., *h* = *h*^1^, *A* = *M* = 1 (hence, also denote *V* = *V*^1^). Similarly to function *g*, the form of function *h* should be related to the structure of the “basic cell” presented in Figure 2b. Thus, the fluctuation shape function can be approximated by the periodic approximation function in the following form:

(24) $$\tilde{h}(x,\bar{x})=l^{2}[\cos(2\pi \bar{x}/l)+c(x)],\;\bar{x}\in \Delta (x),\;x\in \Lambda ,$$

where function *c*(*x*) is a slowly varying one in *x*, determined by $<\tilde{\mu }\tilde{h}>=0$; function $\tilde{\gamma }(x)$ is the periodic approximation of the distribution function γ(*x*). Function *c*(*x*) is treated as constant in calculations of derivatives $\partial \tilde{h},\;\partial \partial \tilde{h}$.

### 7.2. Free Vibration Equations

#### 7.2.1. Case (1): FG Plate Bands with a Span L Along the *x*-Axis

Assuming that the central part of the cells of these bands (cf. Figure 1b and Figure 2b) is the reinforcement or the rib of the plate, it can be observed that this case (1) corresponds to plate bands with reinforcement ribs parallel to the edges of support.

1. FG plate bands with a-type microstructure

Let us introduce some denotations:

(25) $$\begin{array}{l} B_{11}=<b_{1111}>,\;B_{11}^{1}=<b_{1111}\partial g>,\;B_{11}^{11}=<b_{1111}\partial g\partial g>,\;B_{21}^{11}=<b_{2121}\partial g\partial g>,\\ m=<\mu >,\;j=<\vartheta >,\;j^{11}=l^{-2}<\vartheta gg>. \end{array}$$

- The tolerance model for an a-type microstructure

The tolerance model equations of free vibrations for FG plate bands with an a-type microstructure are obtained from (18) with the averaged coefficients (25) in the following form:

(26) $$\begin{array}{l} \partial \partial (B_{11}\partial \partial u+B_{11}^{1}r_{1})+m\ddot{u}-j\partial \partial \ddot{u}=0,\\ B_{11}^{1}\partial \partial u+B_{11}^{11}r_{1}+l^{2}j^{11}{\ddot{r}}_{1}=0,\\ B_{21}^{11}r_{2}+l^{2}j^{11}{\ddot{r}}_{2}=0. \end{array}$$

The above equations describe both *macrovibrations* (related to the averaged macrostructure of the plate band under consideration) and *microvibrations* (related to the plate band microstructure) for two fluctuation amplitudes *r*_α_, α = 1, 2. It can be observed that the equation of *r*_2_ is independent.

- The asymptotic model for an a-type microstructure

For the considered plate band, by applying coefficients (25), the asymptotic model equations of free vibrations for FG plate bands with an a-type microstructure are derived from (19) in the following form:

(27) $$\begin{array}{l} \partial \partial (B_{11}\partial \partial u+B_{11}^{1}r_{1})+m\ddot{u}-j\partial \partial \ddot{u}=0,\\ B_{11}^{1}\partial \partial u+B_{11}^{11}r_{1}=0,\;B_{21}^{11}r_{2}=0. \end{array}$$

Because terms with the microstructure parameter *l* are omitted in the above equations, the effect of the microstructure size is neglected. Equation (27) only describes the macrovibrations of the plate band.

2. FG plate bands with b-type microstructure

Let us introduce some denotations:

(28) $$\begin{array}{l} B_{11}=<b_{1111}>,\;D_{11}^{1}=<b_{1111}\partial \partial h>,\;D_{11}^{11}=<b_{1111}\partial \partial h\partial \partial h>,\\ m=<\mu >,\;j=<\vartheta >,\;m^{11}=l^{-4}<\mu hh>,\;{\bar{j}}^{11}=l^{-2}<\vartheta \partial h\partial h>. \end{array}$$

- The tolerance model for a b-type microstructure

The tolerance model equations of free vibrations for FG plate bands with a b-type microstructure are obtained from (20) with the averaged coefficients (28) in the following form:

(29) $$\begin{array}{l} \partial \partial (B_{11}\partial \partial W+D_{11}^{1}V)+m\ddot{W}-\partial (j\partial \ddot{W})=0,\\ D_{11}^{1}\partial \partial W+D_{11}^{11}V+l^{4}m^{11}\ddot{V}+l^{2}{\bar{j}}^{11}\ddot{V}=0. \end{array}$$

The above equations describe both *macrovibrations* (related to the averaged macrostructure of the plate band under consideration) and *microvibrations* (related to the plate band microstructure).

- The asymptotic model for a b-type microstructure

For the considered plate band, by applying coefficients (28), the asymptotic model equations of free vibrations for FG plate bands with a b-type microstructure are derived from (21) in the following form:

(30) $$\begin{array}{l} \partial \partial (B_{11}\partial \partial W+D_{11}^{1}V)+m\ddot{W}-\partial (j\partial \ddot{W})=0,\\ D_{11}^{1}\partial \partial W+D_{11}^{11}V=0. \end{array}$$

Because terms with the microstructure parameter *l* are neglected in the above equations, the effect of the microstructure size is neglected. Equation (30) only describes the macrovibrations of the plate band.

It should be observed that model Equations (26), (27), (29), and (30) have slowly varying functional coefficients in *x*.

#### 7.2.2. Case (2): FG Plate Bands with a Span L_2_ Along the *y*-Axis

By analogy with Section 7.2.1, it can be seen that case (2) corresponds to plate bands with reinforcement ribs perpendicular to the edges of support.

1. FG plate bands with a-type microstructure

In this case, the following denotations are introduced:

(31) $$\begin{array}{l} B_{22}=<b_{2222}>,\;B_{12}^{1}=<b_{1122}\partial g>,\;B_{11}^{11}=<b_{1111}\partial g\partial g>,\\ B_{21}^{11}=<b_{2121}\partial g\partial g>,\;B_{12}^{11}=<b_{1122}\partial g\partial g>,\\ {\bar{B}}_{12}^{11}=l^{-2}<b_{2121}gg>,\;{\bar{B}}_{22}^{11}=l^{-2}<b_{2222}gg>,\\ m=<\mu >,j=<\vartheta >,\;j^{11}=l^{-2}<\vartheta gg>. \end{array}$$

- The tolerance model for an a-type microstructure

The tolerance model equations of free vibrations for FG plate bands with an a-type microstructure are obtained from (18) with the averaged coefficients (31) in the following form:

(32) $$\begin{array}{l} \bar{\partial }\bar{\partial }(B_{22}\bar{\partial }\bar{\partial }u+B_{12}^{1}r_{1})+m\ddot{u}-j\bar{\partial }\bar{\partial }\ddot{u}=0,\\ B_{12}^{1}\bar{\partial }\bar{\partial }u+B_{11}^{11}r_{1}-l^{2}{\bar{B}}_{12}^{11}\bar{\partial }\bar{\partial }r_{1}+l^{2}j^{11}{\ddot{r}}_{1}=0,\\ B_{21}^{11}r_{2}-l^{2}{\bar{B}}_{22}^{11}\bar{\partial }\bar{\partial }r_{2}+l^{2}j^{11}{\ddot{r}}_{2}=0. \end{array}$$

Equation (32) determines both *macrovibrations* (related to the averaged macrostructure of a considered plate band) and *microvibrations* (related to the plate band microstructure) for macrodeflection *u* and two fluctuation amplitudes *r*_α_, α = 1, 2. It can be observed that the equation of *r*_2_ is independent.

- The asymptotic model for an a-type microstructure

For this plate band, by applying denotations (31), the asymptotic model equations of free vibrations for FG plate bands with an a-type microstructure are derived from (19) as follows:

(33) $$\begin{array}{l} \bar{\partial }\bar{\partial }(B_{22}\bar{\partial }\bar{\partial }u+B_{12}^{1}r_{1})+m\ddot{u}-j\bar{\partial }\bar{\partial }\ddot{u}=0,\\ B_{12}^{1}\bar{\partial }\bar{\partial }u+B_{11}^{11}r_{1}=0,\;\;B_{21}^{11}r_{2}=0. \end{array}$$

Since the coefficients with the microstructure parameter *l* are neglected in (33), then the effect of the microstructure size is omitted in these equations. Equation (33) only describes macrovibrations of the plate band.

2. FG plate bands with b-type microstructure

In this case, the following denotations are used:

(34) $$\begin{array}{l} B_{22}=<b_{2222}>,\;D_{21}^{1}=<b_{2211}\partial \partial h>,\;D_{11}^{11}=<b_{1111}\partial \partial h\partial \partial h>,\;{\bar{D}}_{22}^{1}=l^{-2}<b_{2222}h>,\\ {\tilde{D}}_{22}^{11}=l^{-4}<b_{2222}hh>,\;{\overset{⌣}{D}}_{21}^{11}=l^{-2}<b_{2211}\partial \partial hh>,\;{\bar{D}}_{21}^{11}=l^{-2}<b_{2211}\partial h\partial h>,\\ m=<\mu >,\;j=<\vartheta >,\;m^{11}=l^{-4}<\mu hh>,\\ {\bar{j}}^{1}=l^{-2}<\vartheta h>,\;{\bar{j}}^{11}=l^{-2}<\vartheta \partial h\partial h>,\;{\tilde{j}}^{11}=l^{-4}<\vartheta hh>. \end{array}$$

- The tolerance model for a b-type microstructure

The tolerance model equations of free vibrations for FG plate bands with a b-type microstructure are obtained from (20) with the averaged coefficients (34) in the following form:

(35) $$\begin{array}{l} \bar{\partial }\bar{\partial }(B_{22}\bar{\partial }\bar{\partial }W+D_{21}^{1}V)+m\ddot{W}-\bar{\partial }(j\bar{\partial }\ddot{W})+\\ +l^{2}{\bar{D}}_{22}^{1}\bar{\partial }\bar{\partial }\bar{\partial }\bar{\partial }V-l^{2}{\bar{j}}^{1}\bar{\partial }\bar{\partial }\ddot{V}=0,\\ D_{21}^{1}\bar{\partial }\bar{\partial }W+l^{2}{\bar{D}}_{22}^{1}\bar{\partial }\bar{\partial }\bar{\partial }\bar{\partial }W+D_{11}^{11}V+l^{4}m^{11}\ddot{V}+l^{2}{\bar{j}}^{11}\ddot{V}+\\ +2l^{2}({\overset{⌣}{D}}_{21}^{11}-2{\bar{D}}_{21}^{11})\bar{\partial }\bar{\partial }V+l^{4}{\tilde{D}}_{22}^{11}\bar{\partial }\bar{\partial }\bar{\partial }\bar{\partial }V-l^{2}{\bar{j}}^{1}\bar{\partial }\bar{\partial }\ddot{W}+l^{4}{\tilde{j}}^{11}\bar{\partial }\bar{\partial }\ddot{V}=0. \end{array}$$

Equation (35) determines both *macrovibrations* (related to the averaged macrostructure of the plate band) and *microvibrations* (related to the plate band microstructure).

- The asymptotic model for a b-type microstructure

For the considered plate band, by applying coefficients (34), the asymptotic model equations of free vibrations for FG plate bands with a b-type microstructure derived from (21) take the following form:

(36) $$\begin{array}{l} \bar{\partial }\bar{\partial }(B_{22}\bar{\partial }\bar{\partial }W+D_{21}^{1}V)+m\ddot{W}-\bar{\partial }(j\bar{\partial }\ddot{W})=0,\\ D_{21}^{1}\bar{\partial }\bar{\partial }W+D_{11}^{11}V=0. \end{array}$$

The effect of the microstructure size is neglected in the above equations because there are no terms dependent on the microstructure parameter *l* within them. Hence, Equation (36) describes macrovibrations of the plate band only.

It should be observed that model Equations (32), (33), (35), and (36) have slowly varying functional coefficients in *x*.

### 7.3. Free Vibration Frequencies—The Ritz Method Applied to the Model Equations

In this section, formulas of free vibration frequencies for FG plate with the tolerance-periodic microstructure introduced above are obtained.

Because Equations (26), (27), (29), (30), (32), (33), (35), and (36) have slowly varying functional coefficients, analytical solutions of these equations are difficult to find. Approximate formulas of free vibration frequencies can be obtained by applying, for instance, the known Ritz method, cf. [2,79]. In this method, the relation between the maximal strain energy $E_{\max}$ and the maximal kinetic energy $K_{\max}$ have to be derived.

#### 7.3.1. Case (1): FG Plate Bands with a Span L Along the *x*-Axis

1. FG plate bands with a-type microstructure

Solutions to Equations (26) and (27) can be assumed in the following form:

(37) $$u(x,t)=A_{u}\Phi (kx)\cos(\omega t),\;\;r_{\alpha }(x,t)=A_{r_{\alpha }}\Psi (kx)\cos(\omega t),\;\;\alpha =1,2;$$

where *k* is a wave number; ω is a free vibration frequency; functions Φ(*x*), Ψ(*x*) satisfy boundary conditions for the considered FG plate band with an a-type microstructure, in *x* = 0, *L*; and $A_{u},\;A_{r_{\alpha }},\;\alpha =1,2$ are amplitudes. The derivatives of functions Φ(*x*), Ψ(*x*) are denoted as follows:

(38) $$\begin{array}{ll} \tilde{\Phi }(kx)=k\partial \Phi (kx), & \tilde{\Psi }(kx)=k\partial \Psi (kx),\\ \bar{\Phi }(kx)=k^{2}\partial \partial \Phi (kx), & \bar{\Psi }(kx)=k^{2}\partial \partial \Psi (kx). \end{array}$$

Using functions (37) and (38), and also coefficients (25), the following denotations can be introduced:

(39) $$\begin{array}{lll} B=\int_{0}^{L}B_{11}{\left[\bar{\Phi }(kx)\right]}^{2}dx, & B^{\prime }=\int_{0}^{L}B_{11}^{1}\bar{\Phi }(kx)\Psi (kx)dx, & \\ B^{\prime \prime }=\int_{0}^{L}B_{11}^{11}{\left[\Psi (kx)\right]}^{2}dx, & \hat{B^{\prime \prime }}=\int_{0}^{L}B_{21}^{11}{\left[\Psi (kx)\right]}^{2}dx, & \\ m^{\prime }=\int_{0}^{L}m{\left[\Phi (kx)\right]}^{2}dx, & j^{\prime }=\int_{0}^{L}j{\left[\tilde{\Phi }(kx)\right]}^{2}dx, & j^{\prime \prime }=\int_{0}^{L}j^{11}{\left[\Psi (kx)\right]}^{2}dx. \end{array}$$

Then, the formulas of the maximal energies—strain $E_{\max}$ and kinetic $K_{\max}$—can be derived for both models of FG plates with an a-type microstructure—the tolerance and the asymptotic one.

Using the conditions of the Ritz method in the following form:

(40) $$\frac{\partial (E_{\max}-K_{\max})}{\partial A_{u}}=0,\;\frac{\partial (E_{\max}-K_{\max})}{\partial A_{r_{1}}}=0,\;\frac{\partial (E_{\max}-K_{\max})}{\partial A_{r_{2}}}=0,$$

the following characteristic equations can be derived for these models:

- The tolerance model
  (41) $$\begin{array}{l} l^{2}j^{\prime \prime }(k^{2}j^{\prime }+m^{\prime })\omega^{4}-[k^{4}Bl^{2}j^{\prime \prime }+B^{\prime \prime }(k^{2}j^{\prime }+m^{\prime })]\omega^{2}+k^{4}(BB^{\prime \prime }-{B^{\prime }}^{2})=0,\\ -l^{2}j^{\prime \prime }\omega^{2}+\hat{B^{\prime \prime }}=0; \end{array}$$
- The asymptotic model
  (42) $$-B^{\prime \prime }(k^{2}j^{\prime }+m^{\prime })\omega^{2}+k^{4}(BB^{\prime \prime }-{B^{\prime }}^{2})=0.$$
  Solutions of the above equations stand for the free vibration frequencies for the considered models.

Equation (41) leads to the formulas of the free vibration frequencies for the tolerance model in the following form:

(43) $$\begin{array}{l} \omega_{-,+1}=\sqrt{\begin{array}{l} \frac{k^{4}Bl^{2}j^{\prime \prime }+B^{\prime \prime }(k^{2}j^{\prime }+m^{\prime })}{2l^{2}j^{\prime \prime }(k^{2}j^{\prime }+m^{\prime })}\mp \\ \mp \frac{\sqrt{{\left[k^{4}Bl^{2}j^{\prime \prime }+B^{\prime \prime }(k^{2}j^{\prime }+m^{\prime })\right]}^{2}-4l^{2}j^{\prime \prime }(k^{2}j^{\prime }+m^{\prime })k^{4}(BB^{\prime \prime }-{B^{\prime }}^{2})}}{2l^{2}j^{\prime \prime }(k^{2}j^{\prime }+m^{\prime })} \end{array}},\\ \omega_{+2}=\sqrt{\frac{B^{\prime \prime }}{l^{2}j^{\prime \prime }}}; \end{array}$$

where ω_−_ is *the fundamental lower free vibration frequency* related to the averaged macrostructure of the considered FG plate band; ω_+1_, ω_+2_ are *the higher free vibration frequencies* related to an a-type microstructure of the plate band.

However, only one formula of *the free vibration frequency for the asymptotic model* is obtained from Equation (42):

(44) $$\omega_{0}=\sqrt{\frac{k^{4}(BB^{\prime \prime }-{B^{\prime }}^{2})}{B^{\prime \prime }(k^{2}j^{\prime }+m^{\prime })}},$$

which is *the fundamental lower free vibration frequency* related to the averaged macrostructure of the FG plate band.

It can be observed that in the tolerance model for the considered FG plate bands, only fundamental lower-order vibrations (macrovibrations) and higher-order vibrations (microvibrations) along the *x*-axis are coupled.

2. FG plate bands with b-type microstructure

Solutions to Equations (29) and (30) are assumed in the following form:

(45) $$W(x,t)=A_{W}\Theta (kx)\cos(\omega t),\;V(x,t)=A_{V}\Xi (kx)\cos(\omega t);$$

where *k* is a wave number; ω is a free vibration frequency; functions Θ(*x*), Ξ(*x*) satisfy boundary conditions for the considered FG plate band with a b-type microstructure in *x* = 0, *L*; and $A_{W},\;A_{V}$ are amplitudes. Let derivatives of functions Θ(*x*), Ξ(*x*) be denoted as follows:

(46) $$\begin{array}{ll} \tilde{\Theta }(kx)=k\partial \Theta (kx), & \tilde{\Xi }(kx)=k\partial \Xi (kx),\\ \bar{\Theta }(kx)=k^{2}\partial \partial \Theta (kx), & \bar{\Xi }(kx)=k^{2}\partial \partial \Xi (kx). \end{array}$$

Using functions (45) and (46), and also coefficients (28), let us introduce the following denotations:

(47) $$\begin{array}{lll} B=\int_{0}^{L}B_{11}{\left[\bar{\Theta }(kx)\right]}^{2}dx, & D^{\prime }=\int_{0}^{L}D_{11}^{1}\bar{\Theta }(kx)\Xi (kx)dx=0, & D^{\prime \prime }=\int_{0}^{L}D_{11}^{11}{\left[\Xi (kx)\right]}^{2}dx,\\ m^{\prime }=\int_{0}^{L}m{\left[\Theta (kx)\right]}^{2}dx, & j^{\prime }=\int_{0}^{L}j{\left[\tilde{\Theta }(kx)\right]}^{2}dx, & \\ m^{\prime \prime }=\int_{0}^{L}m^{11}{\left[\Xi (kx)\right]}^{2}dx, & \bar{j^{\prime \prime }}=\int_{0}^{L}{\bar{j}}^{11}{\left[\Xi (kx)\right]}^{2}dx. & \end{array}$$

Then, the formulas of the maximal energies—strain $E_{\max}$ and kinetic $K_{\max}$—can be obtained for both models of FG plates with a b-type microstructure—the tolerance and the asymptotic one.

Applying the conditions of the Ritz method

(48) $$\frac{\partial (E_{\max}-K_{\max})}{\partial A_{W}}=0,\;\frac{\partial (E_{\max}-K_{\max})}{\partial A_{V}}=0,$$

the following characteristic equations are obtained for these models:

- The tolerance model
  (49) $$l^{2}(k^{2}j^{\prime }+m^{\prime })(\bar{j^{\prime \prime }}+l^{2}m^{\prime \prime })\omega^{4}-[k^{4}Bl^{2}(\bar{j^{\prime \prime }}+l^{2}m^{\prime \prime })+D^{\prime \prime }(k^{2}j^{\prime }+m^{\prime })]\omega^{2}+k^{4}(BD^{\prime \prime }-{D^{\prime }}^{2})=0;$$
- The asymptotic model
  (50) $$-D^{\prime \prime }(k^{2}j^{\prime }+m^{\prime })\omega^{2}+k^{4}(BD^{\prime \prime }-{D^{\prime }}^{2})=0.$$
  Formulas of the free vibration frequencies for the considered models are obtained as solutions of Equations (49) and (50).

Hence, the formulas of the free vibration frequencies for the tolerance model take the following form:

(51) $${\tilde{\omega }}_{-,+}=\sqrt{\begin{array}{l} \frac{k^{4}Bl^{2}({\bar{j}}^{\prime \prime }+l^{2}m^{\prime \prime })+D^{\prime \prime }(k^{2}j^{\prime }+m^{\prime })}{2l^{2}({\bar{j}}^{\prime \prime }+l^{2}m^{\prime \prime })(k^{2}j^{\prime }+m^{\prime })}\mp \\ \mp \frac{\sqrt{{\left[k^{4}Bl^{2}({\bar{j}}^{\prime \prime }+l^{2}m^{\prime \prime })+D^{\prime \prime }(k^{2}j^{\prime }+m^{\prime })\right]}^{2}-4l^{2}({\bar{j}}^{\prime \prime }+l^{2}m^{\prime \prime })(k^{2}j^{\prime }+m^{\prime })k^{4}(BD^{\prime \prime }-{D^{\prime }}^{2})}}{2l^{2}({\bar{j}}^{\prime \prime }+l^{2}m^{\prime \prime })(k^{2}j^{\prime }+m^{\prime })} \end{array}};$$

where ${\tilde{\omega }}_{-}$ is *the fundamental lower free vibration frequency* related to the averaged macrostructure of the considered FG plate band; ${\tilde{\omega }}_{+}$ is *the higher free vibration frequency* related to the microstructure of the FG plate band.

Similarly to plates with an a-type microstructure, only one formula of *the free vibration frequency for the asymptotic model* is derived:

(52) $${\tilde{\omega }}_{0}=\sqrt{\frac{k^{4}(BD^{\prime \prime }-{D^{\prime }}^{2})}{D^{\prime \prime }(k^{2}j^{\prime }+m^{\prime })}};$$

This is *the fundamental lower free vibration frequency* related to the averaged macrostructure of the FG plate band.

Moreover, in the tolerance model for the considered FG plate bands with a b-type microstructure, fundamental macrovibrations and microvibrations are coupled.

#### 7.3.2. Case (2): FG Plate Bands with a Span L_2_ Along the *y*-Axis

1. FG plate bands with a-type microstructure

Solutions to Equations (32) and (33) are assumed in the form similar to (37), i.e., as follows:

(53) $$u(y,t)=A_{u}\Phi (ky)\cos(\omega t),\;\;r_{\alpha }(y,t)=A_{r_{\alpha }}\Psi (ky)\cos(\omega t),\;\;\alpha =1,2;$$

where *k* is a wave number; ω is a free vibration frequency; functions Φ(*y*), Ψ(*y*) satisfy boundary conditions for the considered FG plate band with an a-type microstructure, in *y* = 0, *L*_2_; and $A_{u},\;A_{r_{\alpha }},\;\alpha =1,2$ are amplitudes. The first and second derivatives of functions Φ(*y*), Ψ(*y*) are denoted as follows:

(54) $$\begin{array}{ll} \tilde{\Phi }(ky)=k\partial \Phi (ky), & \tilde{\Psi }(ky)=k\partial \Psi (ky),\\ \bar{\Phi }(ky)=k^{2}\partial \partial \Phi (ky), & \bar{\Psi }(ky)=k^{2}\partial \partial \Psi (ky). \end{array}$$

The length of the plate band is assumed as *L*_2_ = *L*. Applying functions (53) and (54) and coefficients (31), the following denotations are introduced:

(55) $$\begin{array}{lll} \hat{B}=\int_{0}^{L}B_{22}{\left[\bar{\Phi }(ky)\right]}^{2}dy, & \hat{B^{\prime }}=\int_{0}^{L}B_{12}^{1}\bar{\Phi }(ky)\Psi (ky)dy, & \\ B^{\prime \prime }=\int_{0}^{L}B_{11}^{11}{\left[\Psi (ky)\right]}^{2}dy, & \hat{B^{\prime \prime }}=\int_{0}^{L}B_{21}^{11}{\left[\Psi (ky)\right]}^{2}dy, & \overset{⌣}{B^{\prime \prime }}=\int_{0}^{L}B_{12}^{11}{\left[\Psi (ky)\right]}^{2}dy,\\ {\bar{B}}^{\prime \prime }=\int_{0}^{L}{\bar{B}}_{12}^{11}{\left[\tilde{\Psi }(ky)\right]}^{2}dy, & \tilde{B^{\prime \prime }}=\int_{0}^{L}{\bar{B}}_{22}^{11}{\left[\tilde{\Psi }(ky)\right]}^{2}dy, & \\ m^{\prime }=\int_{0}^{L}m{\left[\Phi (ky)\right]}^{2}dy, & j^{\prime }=\int_{0}^{L}j{\left[\tilde{\Phi }(ky)\right]}^{2}dy, & j^{\prime \prime }=\int_{0}^{L}j^{11}{\left[\Psi (ky)\right]}^{2}dy. \end{array}$$

Then, the formulas of the maximal energies—strain $E_{\max}$ and kinetic $K_{\max}$—can be obtained for both models of FG plates with an a-type microstructure—the tolerance and the asymptotic one.

Applying the conditions of the Ritz method in the following form:

(56) $$\frac{\partial (E_{\max}-K_{\max})}{\partial A_{u}}=0,\;\frac{\partial (E_{\max}-K_{\max})}{\partial A_{r_{1}}}=0,\;\frac{\partial (E_{\max}-K_{\max})}{\partial A_{r_{2}}}=0,$$

the following characteristic equations are derived for these models:

- The tolerance model
  (57) $$\begin{array}{l} l^{2}j^{\prime \prime }(k^{2}j^{\prime }+m^{\prime })\omega^{4}-[k^{4}\hat{B}l^{2}j^{\prime \prime }+(k^{2}l^{2}{\bar{B}}^{\prime \prime }+B^{\prime \prime })(k^{2}j^{\prime }+m^{\prime })]\omega^{2}+\\ +k^{4}\hat{B}(k^{2}l^{2}{\bar{B}}^{\prime \prime }+B^{\prime \prime })-k^{4}{\left(\hat{B^{\prime }}\right)}^{2}=0,\\ -l^{2}j^{\prime \prime }\omega^{2}+\hat{B^{\prime \prime }}-l^{2}k^{2}\tilde{B^{\prime \prime }}=0; \end{array}$$
- The asymptotic model
  (58) $$-B^{\prime \prime }(k^{2}j^{\prime }+m^{\prime })\omega^{2}+k^{4}(\hat{B}B^{\prime \prime }-{\hat{B^{\prime }}}^{2})=0.$$
  Formulas of the free vibration frequencies for the considered models are obtained as solutions of the above equations.

Solving Equation (57), the formulas of the free vibration frequencies for the tolerance model are derived:

(59) $$\begin{array}{l} {\omega^{\prime }}_{-,+1}=\sqrt{\begin{array}{l} \frac{k^{4}\hat{B}l^{2}j^{\prime \prime }+(k^{2}l^{2}{\bar{B}}^{\prime \prime }+B^{\prime \prime })(k^{2}j^{\prime }+m^{\prime })}{2l^{2}j^{\prime \prime }(k^{2}j^{\prime }+m^{\prime })}\mp \\ \mp \frac{\sqrt{\begin{array}{l} {}[k^{4}\hat{B}l^{2}j^{\prime \prime }+(k^{2}l^{2}{\bar{B}}^{\prime \prime }+B^{\prime \prime })(k^{2}j^{\prime }+m^{\prime })]2-\\ -4l^{2}j^{\prime \prime }(k^{2}j^{\prime }+m^{\prime })k^{4}[\hat{B}(k^{2}l^{2}{\bar{B}}^{\prime \prime }+B^{\prime \prime })-{\left(\hat{B^{\prime }}\right)}^{2}] \end{array}}}{2l^{2}j^{\prime \prime }(k^{2}j^{\prime }+m^{\prime })} \end{array}},\\ {\omega^{\prime }}_{+2}=\sqrt{\frac{\hat{B^{\prime \prime }}-l^{2}k^{2}\tilde{B^{\prime \prime }}}{l^{2}j^{\prime \prime }\omega^{2}}}; \end{array}$$

where *the fundamental lower free vibration frequency* related to the averaged macrostructure of the considered FG plate band is denoted by ${\omega^{\prime }}_{-}$, and *the higher free vibration frequencies* related to the plate band a-type microstructure are denoted by ${\omega^{\prime }}_{+1},\;{\omega^{\prime }}_{+2}$.

But from Equation (58), only one formula of *the free vibration frequency for the asymptotic model* is obtained:

(60) $${\omega^{\prime }}_{0}=\sqrt{\frac{k^{4}(\hat{B}B^{\prime \prime }-{\hat{B^{\prime }}}^{2})}{B^{\prime \prime }(k^{2}j^{\prime }+m^{\prime })}},$$

which is *the fundamental lower free vibration frequency* related to the averaged macrostructure of the FG plate band.

For the considered FG plate bands, the fundamental lower-order vibrations and higher-order vibrations along the *x*-axis are coupled in the tolerance model only, but higher-order vibrations along the *y*-axis are independent of them.

2. FG plate bands with b-type microstructure

Solutions to Equations (35) and (36) are assumed as follows:

(61) $$W(y,t)=A_{W}\Theta (ky)\cos(\omega t),\;\;V(y,t)=A_{V}\Xi (ky)\cos(\omega t);$$

where *k* is a wave number; ω is a free vibration frequency; functions Θ(*y*), Ξ(*y*) satisfy boundary conditions for the considered FG plate band with a b-type microstructure in *y* = 0, *L*_2_; and $A_{W},\;A_{V}$ are amplitudes. Let the first and second derivatives of functions Θ(*y*), Ξ(*y*) be denoted as follows:

(62) $$\begin{array}{ll} \tilde{\Theta }(ky)=k\partial \Theta (ky), & \tilde{\Xi }(ky)=k\partial \Xi (ky),\\ \bar{\Theta }(ky)=k^{2}\partial \partial \Theta (ky), & \bar{\Xi }(ky)=k^{2}\partial \partial \Xi (ky). \end{array}$$

In the following transformations, it is assumed that *L*_2_ = *L*. Using functions (61) and (62) and also coefficients (34), the following denotations are introduced:

(63) $$\begin{array}{l} \hat{B}=\int_{0}^{L}B_{22}{\left[\bar{\Theta }(ky)\right]}^{2}dy,\;\hat{D^{\prime }}=\int_{0}^{L}D_{21}^{1}\bar{\Theta }(ky)\Xi (ky)dy,\;D^{\prime \prime }=\int_{0}^{L}D_{11}^{11}{\left[\Xi (ky)\right]}^{2}dy,\\ \bar{D^{\prime }}=\int_{0}^{L}{\bar{D}}_{22}^{1}\bar{\Theta }(ky)\bar{\Xi }(ky)dy,\;{\bar{D}}^{\prime \prime }=\int_{0}^{L}{\bar{D}}_{21}^{11}{\left[\tilde{\Xi }(ky)\right]}^{2}dy,\\ \tilde{D^{\prime \prime }}=\int_{0}^{L}{\tilde{D}}_{22}^{11}{\left[\bar{\Xi }(ky)\right]}^{2}dy,\;{\overset{⌣}{D}}^{\prime \prime }=\int_{0}^{L}{\overset{⌣}{D}}_{21}^{11}{\left[\tilde{\Xi }(ky)\right]}^{2}dy,\\ m^{\prime }=\int_{0}^{L}m{\left[\Theta (ky)\right]}^{2}dy,\;j^{\prime }=\int_{0}^{L}j{\left[\tilde{\Theta }(ky)\right]}^{2}dy,\;\bar{j^{\prime }}=\int_{0}^{L}{\bar{j}}^{1}\tilde{\Theta }(ky)\tilde{\Xi }(ky)dy,\\ m^{\prime \prime }=\int_{0}^{L}m^{11}{\left[\Xi (ky)\right]}^{2}dy,\;\bar{j^{\prime \prime }}=\int_{0}^{L}{\bar{j}}^{11}{\left[\Xi (ky)\right]}^{2}dy\tilde{j^{\prime \prime }}=\int_{0}^{L}{\tilde{j}}^{11}{\left[\tilde{\Xi }(ky)\right]}^{2}dy. \end{array}$$

Similarly to case (1) for FG plates with a b-type microstructure, the formulas of the maximal energies—strain $E_{\max}$ and kinetic $K_{\max}$—are obtained for both tolerance and asymptotic models.

From the conditions of the Ritz method

(64) $$\frac{\partial (E_{\max}-K_{\max})}{\partial A_{W}}=0,\;\frac{\partial (E_{\max}-K_{\max})}{\partial A_{V}}=0,$$

the following characteristic equations are obtained for these models:

- The tolerance model
  (65) $$\begin{array}{l} l^{2}[(k^{2}j^{\prime }+m^{\prime })({\bar{j}}^{\prime \prime }+l^{2}m^{\prime \prime }-k^{4}l^{2}{\tilde{j}}^{\prime \prime })-k^{4}l^{2}{\bar{j}}^{\prime })]\omega^{4}-\\ -[k^{4}l^{2}\hat{B}({\bar{j}}^{\prime \prime }+l^{2}m^{\prime \prime }-k^{2}l^{2}{\tilde{j}}^{\prime \prime })+(k^{4}l^{4}{\tilde{D}}^{\prime \prime }+D^{\prime \prime })(k^{2}j^{\prime }+m^{\prime })-\\ -2k^{4}l^{2}(k^{2}j^{\prime }+m^{\prime })({\overset{⌣}{D}}^{\prime \prime }-2{\bar{D}}^{\prime \prime })+2k^{4}l^{2}{\bar{j}}^{\prime }({\hat{D}}^{\prime }-k^{2}l^{2}{\bar{D}}^{\prime })]\omega^{2}+\\ +k^{4}[\hat{B}(k^{4}l^{4}{\tilde{D}}^{\prime \prime }+D^{\prime \prime })-2k^{4}l^{2}\hat{B}({\overset{⌣}{D}}^{\prime \prime }-2{\bar{D}}^{\prime \prime })-{\left({\hat{D}}^{\prime }-k^{2}l^{2}{\bar{D}}^{\prime }\right)}^{2}]=0; \end{array}$$
- The asymptotic model
  (66) $$-D^{\prime \prime }(k^{2}j^{\prime }+m^{\prime })\omega^{2}+k^{4}[\hat{B}D^{\prime \prime }-{\left(\hat{D^{\prime }}\right)}^{2}]=0.$$
  Solving Equation (65), the formulas of *the free vibration frequencies for the tolerance model* of the considered FG plate bands are obtained:
  (67) $${\bar{\omega }}_{-,+}=\sqrt{\begin{array}{l} \frac{\begin{array}{l} {}[k^{4}l^{2}\hat{B}({\bar{j}}^{\prime \prime }+l^{2}m^{\prime \prime }-k^{2}l^{2}{\tilde{j}}^{\prime \prime })+(k^{4}l^{4}{\tilde{D}}^{\prime \prime }+D^{\prime \prime })(k^{2}j^{\prime }+m^{\prime })-\\ -2k^{4}l^{2}(k^{2}j^{\prime }+m^{\prime })({\overset{⌣}{D}}^{\prime \prime }-2{\bar{D}}^{\prime \prime })+2k^{4}l^{2}{\bar{j}}^{\prime }({\hat{D}}^{\prime }-k^{2}l^{2}{\bar{D}}^{\prime })] \end{array}}{2l^{2}[(k^{2}j^{\prime }+m^{\prime })({\bar{j}}^{\prime \prime }+l^{2}m^{\prime \prime }-k^{4}l^{2}{\tilde{j}}^{\prime \prime })-k^{4}l^{2}{\bar{j}}^{\prime })]}\mp \\ \mp \frac{\sqrt{\begin{array}{l} {}[[k^{4}l^{2}\hat{B}({\bar{j}}^{\prime \prime }+l^{2}m^{\prime \prime }-k^{2}l^{2}{\tilde{j}}^{\prime \prime })+(k^{4}l^{4}{\tilde{D}}^{\prime \prime }+D^{\prime \prime })(k^{2}j^{\prime }+m^{\prime })-\\ -2k^{4}l^{2}(k^{2}j^{\prime }+m^{\prime })({\overset{⌣}{D}}^{\prime \prime }-2{\bar{D}}^{\prime \prime })+2k^{4}l^{2}{\bar{j}}^{\prime }({\hat{D}}^{\prime }-k^{2}l^{2}{\bar{D}}^{\prime })]{]}^{2}-\\ -4k^{4}[\hat{B}(k^{4}l^{4}{\tilde{D}}^{\prime \prime }+D^{\prime \prime })-2k^{4}l^{2}\hat{B}({\overset{⌣}{D}}^{\prime \prime }-2{\bar{D}}^{\prime \prime })-{\left({\hat{D}}^{\prime }-k^{2}l^{2}{\bar{D}}^{\prime }\right)}^{2}]\times \\ \times l^{2}[(k^{2}j^{\prime }+m^{\prime })({\bar{j}}^{\prime \prime }+l^{2}m^{\prime \prime }-k^{4}l^{2}{\tilde{j}}^{\prime \prime })-k^{4}l^{2}{\bar{j}}^{\prime })] \end{array}}}{2l^{2}[(k^{2}j^{\prime }+m^{\prime })({\bar{j}}^{\prime \prime }+l^{2}m^{\prime \prime }-k^{4}l^{2}{\tilde{j}}^{\prime \prime })-k^{4}l^{2}{\bar{j}}^{\prime })]} \end{array}};$$
  where *the fundamental lower free vibration frequency* related to the averaged macrostructure of the considered FG plate band is denoted by ${\bar{\omega }}_{-}$; but *the higher free vibration frequency* is related to the b-type microstructure of the plate band by ${\bar{\omega }}_{+}$.

However, only one formula of *the free vibration frequency for the asymptotic model* is obtained, similarly to plates with an a-type microstructure:

(68) $${\bar{\omega }}_{0}=\sqrt{\frac{k^{4}[\hat{B}D^{\prime \prime }-{\left(\hat{D^{\prime }}\right)}^{2}]}{D^{\prime \prime }(k^{2}j^{\prime }+m^{\prime })}};$$

which is *the fundamental lower free vibration frequency* related to the averaged macrostructure of the FG plate band.

Moreover, it should be noticed that fundamental macrovibrations and microvibrations in the tolerance model are coupled for the considered FG plate bands with a b-type microstructure.

## 8. Final Remarks

Vibrations of thin functionally graded plates with two kinds of tolerance-periodic (non-periodic) microstructures have been considered in this paper: (a) with an a-type tolerance-periodic microstructure, in which the size of the microstructure is of an order of the plate thickness, *d~l*; (b) with a b-type tolerance-periodic microstructure, having a size of the microstructure higher than the plate thickness, *d* << *l*.

The work is theoretical in nature, and so are the presented simple examples of calculations. It considers the free vibrations of two cases of plate bands, i.e., case (1) spanning along the microstructure (along the *x*-axis); case (2) spanning across the microstructure (along the *y*-axis), simply supported at the ends. This can be illustrated by assuming that the central part of the cells of these bands (cf. Figure 1b and Figure 2b) is the reinforcement—the rib of the plate. Then, the considered case (1) corresponds to bands with reinforcement ribs parallel to the edges of support, while case (2) concerns bands with reinforcement ribs perpendicular to the edges of support.

It is shown that formulas for the free vibration frequencies can be obtained within the derived models (whereby equations are characterised by continuous, smooth, but still functional coefficients) using well-known methods, such as the Ritz method.

From the derived equations describing the free vibrations of the plate bands considered in the example, as well as the formulae for the free vibration frequencies, the following can be seen:

- In the tolerance models for functionally graded plates with a-type or b-type tolerance-periodic microstructures, a single equation of macrovibrations along the *z*-axis is obtained. Additionally, in the model for FG plates with the a-type microstructure, equations of microvibrations along the *x*- and *y*-axes are derived, while for the FG plates with a b-type microstructure the equations of microvibrations occur along the *z*-axis.
- According to the tolerance models for the considered plate bands with an a-type tolerance-periodic microstructure, the fundamental lower-order vibrations (macrovibrations along the *z*-axis), corresponding to the macrostructure, and higher-order vibrations (microvibrations) along the *x*-axis, corresponding to the microstructure, are coupled; meanwhile, higher-order vibrations along the *y*-axis are independent.
- However, for the plate bands with a b-type microstructure, the macrovibrations along the *z*-axis and microvibrations along the *z*-axis are coupled.
- Within the framework of tolerance models for functionally graded plate bands with an a-type tolerance-periodic microstructure, one fundamental lower-order free vibration frequency, corresponding to the macrostructure, and two higher-order free vibration frequencies, corresponding to the microstructure, are obtained.
- However, for FG plate bands with a b-type microstructure, one lower-order free vibration frequency and one higher-order free vibration frequency are obtained.
- In the asymptotic models for all cases of functionally graded plates under consideration, one equation describing macrovibrations along the *z*-axis and one fundamental lower free vibration frequency are obtained.
- In forthcoming papers, the tolerance model equations of the aforementioned plates will be used to investigate a number of additional, more complex, and interesting problems. The obtained calculation results obtained with both tolerance models will be compared with each other. Furthermore, the selected results will be validated using the finite element method.

## Data Availability

The original contributions presented in the study are included in the article, further inquiries can be directed to the corresponding authors.

## List of Basic Symbols

<Λ*_g_*>, <Λ*_h_*>	averaged Lagrangean of plate
<·>	averaging operator
Δ	basic cell
*b*_αβγδ_	bending stiffnesses of plate
∂	derivative of *x*_1_
$\bar{\partial }$	derivative of *x*_2_
∂_α_, ∂_α...δ_≡∂_α_...∂_δ_	derivatives of *x*_α_
γ(*x*)	distribution function of material properties
*r*_α_, *r*; *V^A^*, *V*	fluctuation amplitudes
$g\in FS_{\delta }^{s}(\Gamma ,\Delta ),\;s=1$ $;\;h^{A},h\in FS_{\delta }^{s}(\Gamma ,\Delta ),\;s=2$	fluctuation shape functions
$HO_{\delta }^{s}(\Gamma ,\Delta )$	highly oscillating function
Λ	Lagrangean of plate
*L*	length of the plate band along the *x*_1_- or *x*_2_-axis
*L*_1_, *L*_2_	lengths of the plate along the *x*_1_-, *x*_2_-axis, respectively
*u*, *W*	macrodeflection
μ	mass density of plate
*l*	microstructure parameter
$\tilde{f}$	periodic approximation (of function *f*)
Π	plate midplane
ν	Poisson’s ratio of plate material
ϑ	rotational mass inertia of plate
$SV_{\delta }^{s}(\Gamma ,\Delta )$	slowly varying function
*d*	thickness of plate
δ	tolerance parameter
$TP_{\delta }^{s}(\Gamma ,\Delta )$	tolerance-periodic function
*E*	Young’s modulus of plate material
